# PTEN Mutations Associated with Increased Recurrence and Decreased Survival in Patients with Prostate Cancer Spinal Metastasis

**DOI:** 10.3390/curroncol32060331

**Published:** 2025-06-04

**Authors:** Albert Antar, Yuanxuan Xia, Abdel-Hameed Al-Mistarehi, Pritika Papali, Melanie Alfonzo Horowitz, Shreya Sriram, Shahab Aldin Sattari, Carly Weber-Levine, Sushanth Neerumalla, Benjamin Z. Mendelson, Sang Lee, Kristin J. Redmond, Ali Bydon, Timothy F. Witham, Nicholas Theodore, Daniel Lubelski

**Affiliations:** 1 School of Medicine, Johns Hopkins University, Baltimore, MD 21205, USA; aantar2@jhmi.edu (A.A.); yxia17@jhmi.edu (Y.X.); aalmist1@jh.edu (A.-H.A.-M.); ppapali1@jh.edu (P.P.); malfonz1@jhmi.edu (M.A.H.); ssriram7@jhmi.edu (S.S.); shahabmds_1387@yahoo.com (S.A.S.); cweberl1@jhmi.edu (C.W.-L.); neerumalla.sushanth@gmail.com (S.N.); slee439@jhmi.edu (S.L.); kjanson3@jhmi.edu (K.J.R.); abydon1@jh.edu (A.B.); twitham2@jhmi.edu (T.F.W.); theodore@jhmi.edu (N.T.); 2 School of Medicine, West Virginia University, Morgantown, WV 26506, USA; bzm00001@mix.wvu.edu

**Keywords:** genetic biomarkers, prostate cancer, spine, malignancy, metastasis, mortality, motor, sensory

## Abstract

Introduction: Prostate cancer with spinal metastases (PCSM) is associated with high morbidity and mortality. The impact of biomarkers on the prognosis of spinal metastases, however, remains unclear. Objective: This study explored associations between potential biomarkers, treatment modalities, survival, and neurological outcomes in PCSM patients. Methods: We conducted a retrospective analysis of 68 patients as part of a neurosurgical cohort with PCSM at a comprehensive cancer center from 2013 to 2023, examining the influence of potential biomarkers, treatment modalities, and demographics on prognosis. The primary outcomes were the identification of biomarkers, overall survival (OS) in years, survival after spinal metastasis in years, spinal metastasis recurrence, and postoperative neurological outcomes via Frankel scores. Results: All the patients (n = 68) had adenocarcinoma, and the median age was 69 years. The mortality rate was 66% with a median OS of 6 years. Seventy-two biomarkers were identified. An accelerated failure time model (AFT) showed that radiotherapy to the prostate increased the OS (TR = 1.805, *p* = 0.001), while smoking status (TR = 0.625, *p* < 0.001) and *PTEN* gene mutations (TR = 0.504, *p* = 0.006) were associated with decreased OS. Kaplan–Meier analysis associated *PTEN* mutations with reduced median OS using the Gehan–Breslow–Wilcoxon test (3.50 vs. 9.49 years; *p* = 0.001). *PTEN* mutations were trending towards but were not significant for decreased survival following spinal metastases (2.04 vs. 3.15 years; *p* = 0.08). Both *PTEN* (*p* = 0.02) and *Tumor Protein 53* (*TP53*, *p* = 0.01) mutations were associated with increased spinal metastasis recurrence when analyzed using Fisher’s exact test. No differences were observed in the median OS or survival after spinal metastases among patients with or without androgen receptor splice variant-7 (AR-V7), prostate-specific membrane antigen (PSMA), *TP53*, or other analyzed biomarkers. Similarly, neither age, receipt of chemotherapy, nor radiotherapy to the spine correlated with OS. Only chemotherapy was associated with a decreased postoperative Frankel Score (*p* = 0.002). Conclusions: *PTEN* mutations and smoking status were associated with decreased OS in patients with PCSM. Both *PTEN* and *TP53* mutations were associated with increased spinal metastasis recurrence. Receipt of radiotherapy to the prostate was correlated with prolonged survival, whereas receipt of radiotherapy to the spine was not. Chemotherapy was associated with decreased postoperative neurological outcomes.

## 1. Introduction

Prostate cancer remains the most frequently diagnosed cancer in men worldwide, with an annual incidence of over 1.4 million cases [1]. Early-stage prostate cancer typically has a favorable prognosis due to its slow progression. Yet, survival rates decline significantly once spinal metastasis occurs, rendering treatment options largely palliative [2].

Spinal metastases often lead to morbidity by inducing pathologic fractures, spinal cord compression, and high levels of pain [3]. Associated neurological degeneration and loss of ambulation typically result in an inability to complete activities of daily living [4]. The risk of neurological deterioration underscores the need to identify novel biomarkers that could more accurately predict morbidity and mortality once spinal metastases occur [5].

Recent work has identified genetic mutations that can serve as biomarkers for diagnosing and prognosticating prostate cancer, such as the fusion of *Transmembrane Serine Protease 2* (*TMPRSSS2)* and *E26 Transformation-Specific* (*ETS)* family genes, *MYC* amplification, and alterations in *Phosphatase and Tensin Homolog* (*PTEN)* and *Tumor Protein 53* (*TP53)* [6]. These biomarkers, including mutations in androgen receptor (AR)-regulated genes and the identification of specific markers like Kinesin family member 11 (KIF11), have paved the way for targeted therapies and personalized treatment strategies, particularly for bone metastases [6,7,8]. However, the mutational profiles of prostate cancer with spinal metastasis (PCSM) have not been extensively studied in a neurosurgical context, where the clinical management and treatment strategies are unique.

In this study, we fill this gap by examining the mutational profiles of prostate cancer with spinal metastasis (PCSM), specifically in a neurosurgical cohort, providing novel insights into how these mutations correlate with treatment strategies and clinical outcomes. This work is among the first to explore these associations within this specific cohort, potentially leading to more tailored and effective treatments for patients with PCSM.

## 2. Methods

### 2.1. Study Design and Ethical Considerations

This study has a retrospective design. After obtaining institutional review board (IRB) approval (IRB00378753), we reviewed the medical records of adult patients as part of a neurosurgical cohort with PCSM at a single comprehensive cancer center from 2013 to 2022. The cohort consisted of all patients with spinal metastasis, both oligometastatic and polymetastatic.

### 2.2. Data Extraction and Outcomes of Interest

Data collected from the medical records by the authors included patient demographics, functional neurological outcomes, treatment modalities, mutations from genetic sequencing reports, and protein expression from immunohistochemistry data contained in pathology reports. Biomarker data consisted of spine metastasis resection pathology analysis. Each patient was retrospectively analyzed to assess whether next-generation sequencing (NGS) information, performed by a third party at the time of primary cancer diagnosis, was available. If so, sequencing data was obtained in the form of sequencing reports, which contained information on biomarkers of clinical interest and biomarkers with unknown significance. The same methods were used to assess immunohistochemistry data from pathology reports. All the biomarkers included in the reports were listed, though only biomarkers with at least five occurrences were incorporated into our statistical analysis.

The demographic variables included smoking status and age at time of surgical intervention, or if no surgical intervention was performed, at diagnosis. Surgical data included spinal procedure(s) performed and associated complications. Functional neurological outcomes included postoperative changes in Frankel scores (Grade A–E) and ambulatory status, as compared to the associated preoperative status [9]. Treatment modalities consisted of chemotherapy, radiotherapy (RT), immunotherapy, and targeted therapy received at any point during the disease course.

Outcomes of interest included the identification of potential biomarkers, overall survival (OS) in years, survival time after first spinal metastases in years, spinal metastasis recurrence, and postoperative neurological outcomes as assessed by Frankel scores. The OS was defined as the time from the initial diagnosis of prostate cancer until death. Survival time after the first spinal metastasis was defined as the period from the initial occurrence of prostate cancer metastasis to the spine until death. Spinal metastasis recurrence was defined as radiographic evidence of spinal metastasis recurrence after initial surgical intervention.

### 2.3. Statistical Analysis

The data were collected using Excel (Microsoft Corp., Redmond, WA, USA) and analyzed using Prism 10 (GraphPad, Boston, MA, USA) and R Studio 4.3.0 (RStudio Inc., Boston, MA, USA). The continuous variables are reported as the median and interquartile range (IQR), while the categorical data are presented as counts and percentages. Kaplan–Meier curves were generated to assess the OS and survival time after spinal metastases. Comparisons were made using the Wilcoxon–Breslow–Gehan test, with a significance level set at 0.05. Patients with biomarkers or who received certain treatments were compared to their counterparts without the same biomarkers or treatment status. An accelerated failure time (AFT) model was developed using stepwise backward selection to explore the associations between potential biomarkers with at least five observations, treatment modalities, and demographics with OS. The AFT model included variables such as age, smoking status, treatment modality, and biomarkers with at least five observations that met a *p*-value of <0.2 in univariate Kaplan–Meier analysis. Time ratios (TR), standard errors (SE), and *p*-values were reported. Time ratios (TR) greater than 1 indicate that the event of interest took longer to occur compared to the specified reference group, and vice versa. Additionally, an ordinal logistic regression model was employed to identify significant predictors of the postoperative Frankel score. The covariates included in the analysis were biomarkers with at least five observations, treatment modalities, smoking status, and preoperative Frankel score. The model generated odds ratios, 95% confidence intervals (CI), and *p*-values, with a significance level set at 0.05. Spinal metastasis recurrence was analyzed for all potential biomarkers with at least five observations using Fisher’s exact test with a significance level set at 0.05. A multiple comparison adjustment was not performed given the size of the dataset and the exploratory nature of the analysis.

## 3. Results

### 3.1. Baseline Characteristics

We identified sixty-eight eligible patients with prostatic adenocarcinoma metastasis to the spine in our dataset, all of whom were included in our study (Table 1). The median age was 69 years, with an IQR of 63–75 years. The follow-up period for each patient extended from the time of initial prostate cancer diagnosis until death or the last recorded encounter.

**Figure 1:**
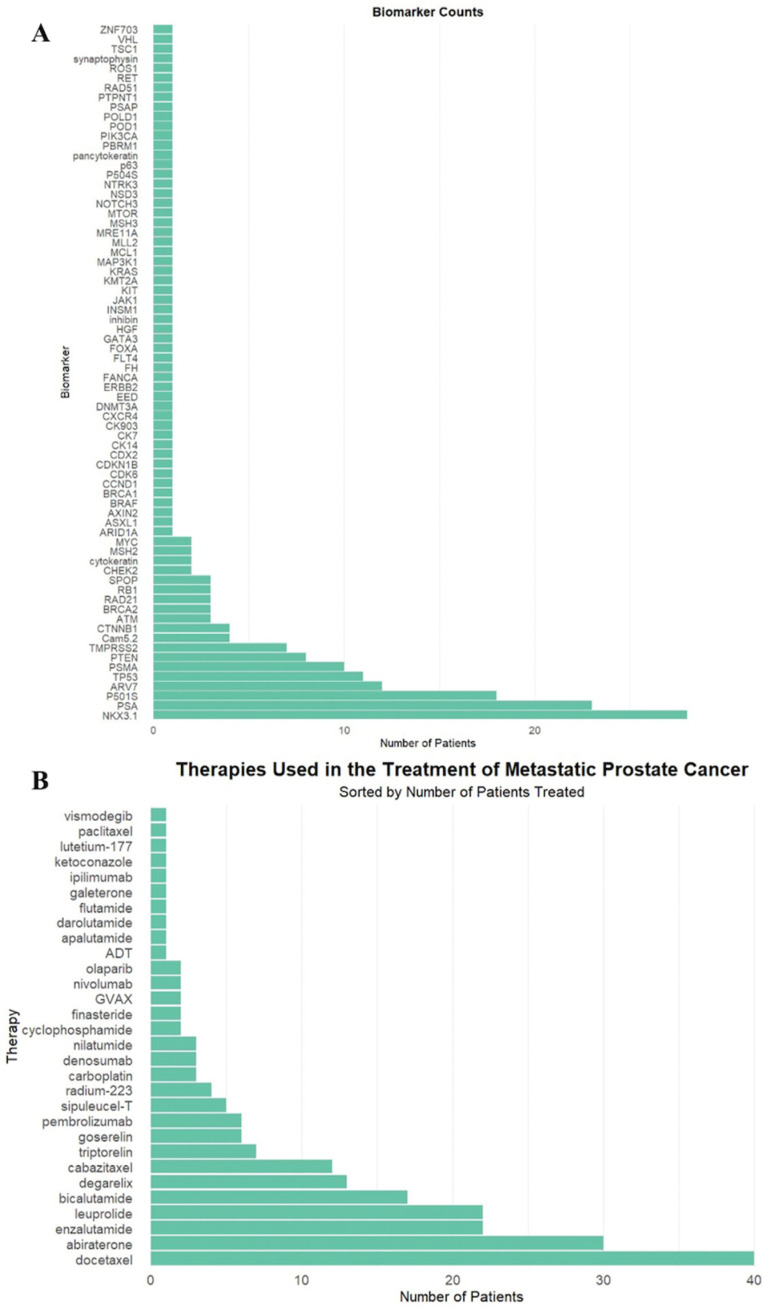
(**A**) Biomarker frequency among all patients in the study population. (**B**) Frequency of therapeutic agents administered to patients in the study population.

In total, 72 unique biomarkers were identified (Figure 1A). These biomarkers were deemed clinically significant due to their inclusion in the pathology and genetic sequencing reports, retrospectively reviewed by authors in patient electronic medical records, as biomarkers of interest.

The most commonly used therapeutics were docetaxel (40, 59%), abiraterone (30, 44%), and enzalutamide (22, 32%), as demonstrated in Figure 1B. Twenty-three patients (34%) received RT targeted at the primary tumor site, and 59 patients (87%) received RT to the spine.

Sixty-two patients received surgical intervention (91%). Of these patients, the most common surgical interventions were laminectomy (52, 84%), fusion (39, 63%), and corpectomy (10, 16%). The vast majority of patients had no surgical complications (59, 95%). Table 1 lists the complications for the remaining patients.

Metastasis included sites other than the osseous spine, listed in Table 1. The predominant levels of spine metastasis were thoracic (29, 68%), lumbar (10, 24%), cervical (3, 6%), and sacral (1, 3%). The most common (mode) preoperative and postoperative Frankel score was D. The differences in the preoperative and postoperative ambulatory status were not significant (*p* = 0.81) (Table 1).

### 3.2. Survival Analysis

The median OS was 6.44 years, with an IQR of 3.32 to 10.82 years. The median time to spinal metastasis was 4.83 years, with an IQR of 2.33 to 9.37 years. Following the development of spinal metastases, the median OS was 2.28 years, with an IQR of 1.04 to 4.53 years.

**Figure 2:**
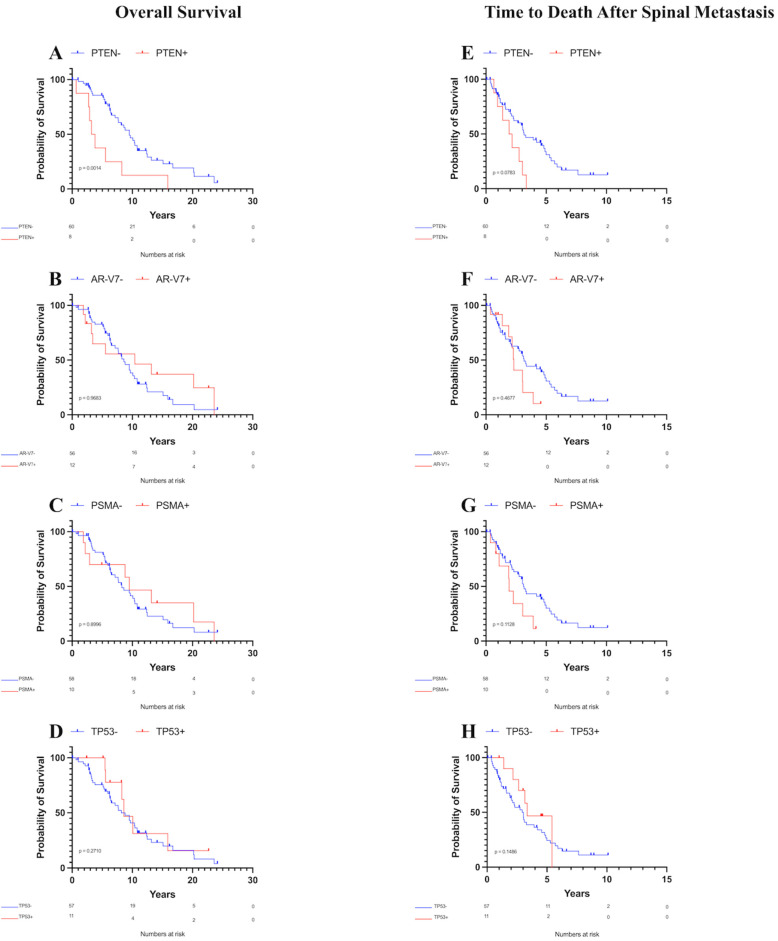
Kaplan–Meier overall survival and survival after spinal metastasis curves for patients with metastatic prostate cancer to the spine. Only PTEN was associated with decreased OS. (**A**) OS by PTEN status, (**B**) OS by AR-V7 status, (**C**) OS by PSMA status, (**D**) OS by TP53 status, (**E**) Survival time after spinal metastasis by PTEN status, (**F**) Survival time after spinal metastasis by AR-V7 status, (**G**) Survival time after spinal metastasis by PSMA status, (**H**) Survival time after spinal metastasis by TP53 status.

Patients with *PTEN* mutations exhibited a decreased median OS of 3.50 years compared to 9.49 years for those without *PTEN* mutations (Figure 2A, *p* = 0.001). Conversely, no significant differences in the median OS were observed between patients with or without androgen receptor splice variant-7 (AR-V7, Figure 2B, *p* = 0.97), prostate-specific membrane antigen (PSMA, Figure 2C, *p* = 0.90), *TP53* mutations (Figure 2D, *p* = 0.27), or any other analyzed biomarker with at least five observations. Furthermore, age at time of surgical intervention (or at diagnosis for patients who did not receive surgical intervention) did not significantly affect the OS according to Kaplan–Meier survival analysis (*p* = 0.08).

Analysis of survival time after spinal metastasis revealed that patients with *PTEN* mutations had a median OS of 2.04 years compared to 3.15 years for those without *PTEN* mutations. This difference was not significant using the Gehan–Breslow–Wilcoxon test, although it was trending towards significance (Figure 2E, *p* = 0.08). No significant differences in the median OS after spinal metastasis were found between patients with or without AR-V7 (Figure 2F, *p* = 0.47), PSMA (Figure 2G, *p* = 0.11), *TP53* mutations (Figure 2H, *p* = 0.15), or any other analyzed biomarker with at least five observations. Additionally, age showed no association with OS after spinal metastasis according to Kaplan–Meier survival analysis (*p* = 0.59).

Finally, spinal metastasis recurrence was analyzed using Fisher’s exact test. In total, 11 (18%) patients experienced spinal metastasis recurrence after surgical intervention, of which 5 (45%) occurred at the same spinal level, 4 (36%) occurred at an adjacent level, and 8 (73%) occurred at a distant level. Both *PTEN* (*p* = 0.02) and *TP53* mutations (*p* = 0.01) were associated with increased spinal metastasis recurrence (Table 2).

### 3.3. Predictive Models

An ordinal logistic regression model was utilized to assess the impact of various factors on the postoperative Frankel score, considering the preoperative Frankel score as a covariate (Table 3). The factors included age, smoking status, treatment modality, and biomarkers with at least five observations. Notably, treatment with chemotherapy was associated with a decreased postoperative Frankel score (*p* = 0.002). Additionally, an accelerated failure time (AFT) model employing stepwise backward selection was applied to explore the influence of factors such as age, smoking status, treatment modality, and biomarkers with at least five observations and *p* < 0.2 from the Wilcoxon–Breslow–Gehan test during Kaplan–Meier analysis on the OS. RT to the prostate was associated with increased OS (Table 4, TR = 1.805, *p* = 0.001). Conversely, smoking status (TR = 0.625, *p* < 0.001) and *PTEN* gene mutations (TR = 0.504, *p* = 0.006) were negatively associated with OS. Table 5 summarizes the major findings.

## 4. Discussion

Metastatic prostate cancer (mPC) is common, with almost one-fifth of patients presenting with metastasis at diagnosis [10]. Prostate cancer has a predilection for osseous metastases, of which the spine is the most common site [2]. Biomarkers offer a novel avenue for the prognostication of morbidity and mortality in patients with PCSM.

### 4.1. Biomarkers

To the best of our knowledge, our results are the first to demonstrate that mutations in *PTEN*, a tumor-suppressor gene, are associated with decreased OS or increased spinal metastasis recurrence in a surgical cohort consisting entirely of prostate cancer patients who developed spinal metastases. Prior research has identified *PTEN* as a gene inactivated by mutations or deletions in patients with prostate cancer [6]. Yoshimoto et al. demonstrated *PTEN* to be associated with a higher Gleason Score and tumor stage, indicating a higher-grade tumor and more aggressive disease course [11]. Ferraldeschi et al. demonstrated that *PTEN* inactivation is associated with worse OS in metastatic castration-resistant prostate cancer (mCRPC) [12]. Likewise, Zhang et al. found *PTEN* mutations to be associated with decreased progression-free survival (PFS) and OS in a group of 205 patients with metastatic castration-naïve prostate cancer (mCNPC) [13]. Further research should explore the potential prognostic value of identifying *PTEN* mutations in patients with PCSM and any associations between *PTEN* mutations and an increased risk of metastasis to other locations.

Our results also demonstrated no difference in OS or survival after spinal metastases in patients with mutations in AR-V7, PSMA, or *TP53*. AR-V7 is an androgen receptor splice variant that is hypothesized to be a potential molecular contributor to prostate cancer progression, given its role in conferring resistance to enzalutamide and abiraterone in preclinical studies [14]. Antonarakis et al. illustrated that patients with the AR-V7 mCRPC variant had shorter PFS and no appreciable clinical benefit with the administration of abiraterone/enzalutamide compared to their counterparts without AR-V7 [15].

The expression of prostate-specific membrane antigen (PSMA), which is upregulated in prostate cancer, has been studied as a prognostic factor. Sweat et al. analyzed 232 patients with node-positive prostatic adenocarcinoma and noted that PSMA expression is significantly greater in prostate adenocarcinoma and lymph node metastases compared to benign prostatic tissue [16]. Likewise, Perner et al. demonstrated that high PSMA levels are associated with increased rates of prostate cancer recurrence [17]. In contrast, Pomykala et al. suggest that PSMA identification does not significantly influence mPC management [18].

Finally, mutations in *TP53*, a tumor-suppressor gene, have been postulated to be associated with decreased survival in patients with prostate cancer. Zhang et al. demonstrated that *TP53* mutations in circulating tumor DNA of Chinese patients with prostate cancer were associated with a higher rate of metastases and castration resistance, in addition to decreased PFS [13]. Zhou et al. identified *TP53* mutations as a negative independent predictor in mPC, especially when co-occurring with other genetic alterations, such as AR-amplification and *PTEN* deletion [19]. Our data suggests that *TP53* mutations may be associated with increased risk of spinal metastasis recurrence.

Therefore, since our results demonstrate no correlation between survival and AR-V7, PSMA, or *TP53* mutation status, this likely reflects the limited statistical power of our study based on our sample size. This is especially true regarding AR-V7 and TP53, whose statuses as negative prognostic factors are better established in the literature.

### 4.2. Treatments

Our analysis revealed that prostate-directed radiation therapy (RT) was associated with improved OS. Mohiuddin et al. concluded that external beam radiation therapy (EBRT) provided a survival benefit when combined with androgen deprivation therapy (ADT) in high-risk prostate cancer patients compared to conservative management [20]. Zelefsky et al. demonstrated in a cohort of 2047 patients with clinically localized prostate cancer that high-dose intensity modulated radiation therapy (IMRT) improved biochemical control and decreased the risk of distant metastases [21].

In contrast, we found that radiation therapy of the spine was not associated with improved OS. While this may represent a true lack of association, it may also represent the limited power of the study based on the sample size. Fischer-Valuck et al. noted improved OS in patients with prostate cancer and bone (but not exclusively spine) metastasis receiving long-course radiation therapy (LC-RT) but not short-course radiation therapy (SC-RT) [22]. Abugharib et al. found spine-directed stereotactic body radiation therapy (SBRT) to be associated with improved 1- and 2-year OS rates in a hormone-sensitive prostate cancer cohort (98% and 95%) compared to a castration-resistant prostate cancer cohort (79% and 65%) [23]. Still, the literature suggests that the timing and staging of prostate cancer are critical in determining the survival benefits of RT, as patients with a higher disease volume burden typically fare worse following RT administration [24].

In comparison to RT, chemotherapy was not associated with OS but was correlated with worse postoperative neurological outcomes. Originally used as a palliative measure, chemotherapy in prostate cancer has evolved to offer survival advantages, largely due to the implementation of docetaxel [25]. Clinical trials suggest that docetaxel is mainly beneficial in lowering prostate-specific antigen (PSA) values but may offer increased survival in patients with prostate cancer early in their disease course [25]. One clinical trial found no significant survival benefit in mCRPC patients, whereas others reported an increase in OS in mCNPC [26,27,28]. Despite these benefits, chemotherapeutic agents are associated with gastrointestinal and cardiovascular toxicities, thromboembolic events, infections, and neuropathy in patients with prostate cancer [25]. Consequently, their side effect profiles may contribute to worse postoperative neurological outcomes with prolonged use. Chemotherapy is also not a first-line treatment for prostate cancer but is, instead, reserved for advanced cases. The observed decline in neurological outcomes among patients with PCSM receiving chemotherapy may, therefore, be more directly attributable to the extent of metastasis rather than the impact of chemotherapy itself.

Finally, our study did not find a survival benefit associated with the use of immunotherapy for patients with prostate cancer and spinal metastases. Again, this may represent a lack of true association or the limited power of the study to detect a true correlation given the sample size. Immunotherapy, which has been found to improve OS in patients with prostate cancer, is an ideal treatment modality as prostate cancer cells express unique proteins, such as PSA and PSMA, that can act as therapeutic targets for the immune system [29]. However, due to the heterogeneity found within the tumor microenvironment in prostate cancer, establishing a standard immunotherapy treatment has been difficult [29]. In mCRPC patients, Sipuleucel-T, an immunotherapy vaccine for prostate cancer, was found to improve OS, though not the PFS or PSA value [30]. Consequently, National Comprehensive Cancer Center Network (NCCN) guidelines recommend using Sipuleucel-T early in the disease course when the immune system retains higher functionality. This is in contrast to the lower immune system functionality observed in mPC patients, as may have been the case in our patient population [31].

### 4.3. Demographic Factors

Our results demonstrated no survival difference based on the age of PCSM patients at the time of surgical intervention or diagnosis. Hamsta et al. reported that younger individuals (less than age 70) are diagnosed with metastatic lesions in prostate cancer at a higher rate when compared to geriatric patients [32]. Although these tumors may present more aggressively in younger patients, this population may be offered more aggressive treatment options [32]. Studies have also linked older age to biases in oncological treatments, which have, at times, resulted in under-treatment [33]. As a result, elderly patients may not be offered more aggressive therapeutic regimens and, subsequently, fare worse. In addition, older age is linked to a higher comorbidity burden, which is often associated with worse outcomes.

Finally, our data indicate that smoking history is associated with decreased OS. This is in line with Foerster et al., who found smoking status to be associated with a higher risk of recurrence, metastasis, and reduced survival in patients with prostate cancer [34]. Kenfield et al. also recognized that smoking status at the time of prostate cancer diagnosis is associated with increased overall mortality, cardiovascular-disease-specific mortality, and prostate-cancer-specific mortality [35]. Smokers are reported to undergo routine PSA testing less frequently and may, as a result, be more likely to be diagnosed at a later stage, increasing their risk of mortality [36]. In addition, Plaskon et al. observed a dose–response relationship between pack-years and prostate cancer risk in middle-aged men, suggesting that smoking may directly influence tumor growth [37].

### 4.4. Limitations

While our study outlines several factors associated with survival and postoperative neurological outcomes in PCSM, it is not without limitations. The scope of our analysis was constrained by a relatively small patient cohort sourced from a single institution, thereby limiting our statistical power. We also combined our analysis to include both protein expression from immunohistochemical data and genetic mutations from sequencing data in an exploratory fashion, which limited our ability to comprehensively analyze each individually. In addition, the retrospective nature of our study allowed us to demonstrate correlation but not causation. Moreover, the small sample size prevented the analysis of specific gene mutations and differences in the chemotherapy and RT received. Therefore, larger multi-institutional studies are needed to better understand how genetic mutations and other potential biomarkers, treatment modalities, and demographic factors affect survival and postoperative neurological outcomes in PCSM patients. Larger studies would also aid in investigating the relationship between primary tumor biomarkers and biomarkers from the resected spinal cord metastatic tumor tissue, and understanding the relationships and interactions between different mutations. Lastly, a homogenous patient population was used, neurosurgical PCSM patients, which may reduce the general validity of our results. Further, large institutional studies not limited to solely neurosurgical PCSM patients are warranted.

## 5. Conclusions

The use of mutation status may aid in the prognostication of morbidity and mortality in patients with PCSM. This study provides novel insights into how specific genetic mutations, particularly PTEN and TP53 mutations, influence clinical outcomes in this unique patient population. In this study, we demonstrated that PTEN mutations and smoking status were associated with decreased overall survival (OS) in PCSM patients. These findings suggest that PTEN mutations, along with smoking history, could be critical factors in early risk stratification. Both PTEN and TP53 mutations were associated with increased spinal metastasis recurrence, highlighting the potential of these mutations as biomarkers for recurrence risk in PCSM patients. Further analyses with larger sample sizes for sufficient power should be conducted to identify specific PTEN mutations associated with poor survival outcomes.

Additionally, our study presents new evidence that radiation therapy (RT) to the prostate was correlated with enhanced survival, whereas RT to the spine did not show the same benefit, suggesting the need for more targeted radiation strategies in the management of spinal metastases. Only chemotherapy was associated with decreased postoperative neurological outcomes, underscoring the complex interplay between systemic treatments and neurological health in this cohort.

By elucidating these associations, our work offers novel insights that could guide clinicians in developing more personalized treatment plans for patients with PCSM, ultimately improving clinical decision-making and patient outcomes. These findings provide a foundation for future studies exploring targeted therapies tailored to the mutational profiles of PCSM patients.

## Figures and Tables

**Table 1:** Demographic and clinical characteristics of study population (n = 68). * Represents other metastatic lesions discovered at the time of spinal metastases. ** Represents top 10 most frequently prescribed treatments.

Parameter	Description
Age	Median: 69 years (63–75)
Smoking status	Never smoked: 35 (52%), Former smokers: 27 (40%), Current smokers: 6 (9%)
Cancer subtype	Adenocarcinoma: 68 (100%)
Mortality	45 out of 68 patients (66%)
Overall survival	Median: 6.44 years (3.32–10.82)
Time to spine metastasis	4.83 years (2.33–9.37)
Overall survival post-spinal metastasis	Median: 2.28 years (1.04–4.53)
Spine level metastasis	Cervical: 6%, Thoracic: 68%, Lumbar: 24%, Sacral: 3%
Other metastasis *	Spine and prostate only: 42 (62%), Diffuse sclerotic bone lesions: 12 (18%), Lung: 8 (12%), Liver: 7 (10%), Pelvis: 6 (9%), Adrenal gland: 3 (4%), Lymph node: 3 (4%), Sternum: 2 (3%), Others (humerus/rib/femur): 1 each (1.5%)
Preop Frankel	Mode: E
Postop Frankel	Mode: E
Preop ambulatory status	Ambulatory: 53 (86%), Non-ambulatory: 9 (14%)
Postop ambulatory status	Ambulatory: 52 (84%), Non-ambulatory: 10 (16%)
Treatment **	Chemotherapy: docetaxel: 40 (59%), cabazitaxel: 12 (18%); Immunotherapy: pembrolizumab: 6 (9%); Targeted therapy: abiraterone: 29 (42.6%), enzalutamide: 20 (29.4%), leuprolide: 19 (27.9%), bicalutamide: 17 (25%), degarelix: 13 (19%), triptorelin: 7 (10%), goserelin: 6 (9%); Radiotherapy: to prostate: 23 (34%), to spine met: 59 (87%)
Postop complications	No complications: 59 (95%), PE: 1 (1.6%), Bowel incontinence: 1 (1.6%), Ileus: 1 (1.6%), Hyponatremia: 1 (1.6%)

**Table 2:** Fisher’s exact test was conducted to correlate spinal metastasis recurrence with biomarker status. Both PTEN and TP53 were associated with recurrence at a significance level of 0.05.

Biomarker	Number of Recurrences	Fisher *p*-Value
AR-V7	3	0.395
NKX3.1	5	0.751
P501S	2	0.714
PSA	3	0.738
PSMA	0	0.197
PTEN	4	0.020
TP53	5	0.012

**Table 3:** An ordinal logistic regression model (backwards selection) was performed to predict the postoperative Frankel Score using biomarkers (n ≥ 5) and various factors, with the preoperative Frankel Score acting as a covariate. Age, radiotherapy to spine and radiotherapy to prostate, immunotherapy, and targeted therapy were included in the original model and eliminated through backwards selection. The preoperative Frankel score and chemotherapy use were associated with predicting the postoperative Frankel score at a 0.05 significance level.

Variable	No. of Patients	Odds Ratio	Std. Error	*p*-Value
Preop Frankel	-	2.373	0.406	0.033
Current smoker status	6	0.221	0.875	0.085
Chemotherapy	38	0.151	0.617	0.002
ARV7	11	3.662	0.763	0.089
PSA	23	2.092	0.549	0.179

**Table 4:** An accelerated failure time model (backwards selection) was used to assess the influence of biomarkers (n ≥ 5) and various covariates on the overall survival. Age, chemotherapy, and radiotherapy to spine were included in the initial model but were found to be insignificant and removed through backward selection. Smoking status, radiotherapy to prostate, and PTEN were associated with overall survival at a 0.05 significance level.

Parameter	Time Ratio	Std. Error	Sig.
Smoking status	0.625	0.130	<0.001
Radiotherapy to prostate	1.805	0.175	0.001
Immunotherapy	0.677	0.201	0.052
Targeted therapy	0.673	0.216	0.066
PTEN	0.504	0.248	0.006

**Table 5:** Summary of major findings.

**Predictors of Survival**		
**Characteristic**	**Effect**	**Time Ratio (Standard Error)**
PTEN mutation	Worse overall survival	0.504 (0.248)
Smoking	Worse overall survival	0.625 (0.130)
Radiotherapy to prostate	Improved overall survival	1.805 (0.175)
**Predictors of Recurrence**		
**Characteristic**	**Effect**	**Number of Recurrences (*p*-value)**
PTEN	Increased recurrences	4 (0.020)
TP53	Increased recurrences	5 (0.012)
**Predictors of Postoperative Frankel Score**		
**Characteristic**	**Effect**	**Odds Ratio (Standard Error)**
Increased preoperative Frankel	Increased postoperative Frankel score	2.373 (0.406)
Chemotherapy	Decreased postoperative Frankel score	0.151 (0.617)

## Data Availability

The datasets generated and analyzed during the current study are available from the corresponding author upon request.

## Abbreviations

PCSM	Prostate cancer with spinal metastasis
IRB	Institutional review board
RT	Radiotherapy
OS	Overall survival
IQR	Interquartile range
AFT	Accelerated failure time
TR	Time ratio
SE	Standard error
CI	Confidence interval
AR-V7	Androgen receptor splice variant-7
PSMA	Prostate-specific membrane antigen
mPC	Metastatic prostate cancer
mCRPC	Metastatic castration-resistant prostate cancer
PFS	Progression-free survival
mCNPC	Metastatic castration-naïve prostate cancer
EBRT	External beam radiation therapy
ADT	Androgen deprivation therapy
IMRT	Intensity modulated radiation therapy
LC-RT	Long-course radiation therapy
SC-RT	Short-course radiation therapy
SBRT	Stereotactic body radiation therapy
PSA	Prostate-specific antigen
NCCN	National Comprehensive Cancer Center Network
